# Developing stem cell therapy for retinal dystrophies

**DOI:** 10.1186/2046-2530-4-S1-O19

**Published:** 2015-07-13

**Authors:** JC Sowden

**Affiliations:** 1 UCL Institute of Child Health, Stem Cells and Regenerative Medicine Section, Birth Defects Research Centre, University College London, London, UK

## 

Inherited retinal disease leading to death of the photoreceptor cells affects around 1 in 3000 people and includes the retinal ciliopathies which disrupt the function of proteins associated with the photoreceptor cilia. We are interested in the potential for repair of the retina and preservation of sight via cell transplantation. This approach could be broadly applicable across a range of inherited retinal dystrophies. Our work has identified an optimal donor cell population from the developing retina, that following subretinal transplantation in adult mice generates large numbers of new functional rod photoreceptors [1]. Although retinal neurogenesis ceases soon after birth, successful integration and differentiation of new rod cells can be achieved within the adult retina by transplanting immature post mitotic cells already committed to a photoreceptor fate. This same type of photoreceptor precursor cell can be isolated from 3D embryonic stem cell-derived retinal differentiation cultures and successfully transplanted into mouse models of retinal disease [2]. Thus, pluripotent stem cell lines offer the potential to generate unlimited quantities of photoreceptor precursor cells for retinal repair and restoration of vision. Current and future challenges for the development of photoreceptor cell replacement therapies for retinal disease will be discussed.
